# Sulforaphane Bioavailability in Healthy Subjects Fed a Single Serving of Fresh Broccoli Microgreens

**DOI:** 10.3390/foods12203784

**Published:** 2023-10-15

**Authors:** John A. Bouranis, Carmen P. Wong, Laura M. Beaver, Sandra L. Uesugi, Ethan M. Papenhausen, Jaewoo Choi, Edward W. Davis, Adilson Nunes Da Silva, Newton Kalengamaliro, Rekha Chaudhary, Jordan Kharofa, Vinita Takiar, Thomas J. Herzog, William Barrett, Emily Ho

**Affiliations:** 1 Linus Pauling Institute, Oregon State University, Corvallis, OR 97331, USA; bouranij@oregonstate.edu (J.A.B.); carmen.wong@oregonstate.edu (C.P.W.); laura.beaver@oregonstate.edu (L.M.B.); sandra.uesugi@oregonstate.edu (S.L.U.); papenhae@oregonstate.edu (E.M.P.); jaewoo.choi@oregonstate.edu (J.C.); 2 School of Public Health and Nutrition, Oregon State University, Corvallis, OR 97331, USA; 3 Center for Quantitative Life Sciences, Oregon State University, Corvallis, OR 97331, USA; ed@cqls.oregonstate.edu; 4 80 Acres Farms, Hamilton, OH 45011, USA; adilson.nunes@eafarms.com (A.N.D.S.); newton.kalengamaliro@infinite-acres.com (N.K.); 5 Department of Medical Oncology, University of Cincinnati, Cincinnati, OH 45221, USA; tripatrr@ucmail.uc.edu; 6 Department of Radiation Oncology, University of Cincinnati, Cincinnati, OH 45221, USA; kharofjr@ucmail.uc.edu (J.K.); takiarva@ucmail.uc.edu (V.T.); barretwl@ucmail.uc.edu (W.B.); 7 Department of OB/GYN, Division of Gynecologic Oncology, University of Cincinnati, Cincinnati, OH 45221, USA; herzogtj@ucmail.uc.edu

**Keywords:** broccoli microgreens, cruciferous vegetables, microbiome, sulforaphane, sulforaphane-nitrile

## Abstract

Cruciferous vegetable consumption is associated with numerous health benefits attributed to the phytochemical sulforaphane (SFN) that exerts antioxidant and chemopreventive properties, among other bioactive compounds. Broccoli sprouts, rich in SFN precursor glucoraphanin (GRN), have been investigated in numerous clinical trials. Broccoli microgreens are similarly rich in GRN but have remained largely unexplored. The goal of this study was to examine SFN bioavailability and the microbiome profile in subjects fed a single serving of fresh broccoli microgreens. Eleven subjects participated in a broccoli microgreens feeding study. Broccoli microgreens GRN and SFN contents and stability were measured. Urine and stool SFN metabolite profiles and microbiome composition were examined. Broccoli microgreens had similar GRN content to values previously reported for broccoli sprouts, which was stable over time. Urine SFN metabolite profiles in broccoli microgreens-fed subjects were similar to those reported previously in broccoli sprouts-fed subjects, including the detection of SFN-nitriles. We also reported the detection of SFN metabolites in stool samples for the first time. A single serving of broccoli microgreens did not significantly alter microbiome composition. We showed in this study that broccoli microgreens are a significant source of SFN. Our work provides the foundation for future studies to establish the health benefits of broccoli microgreens consumption.

## 1. Introduction

**Figure 1:**
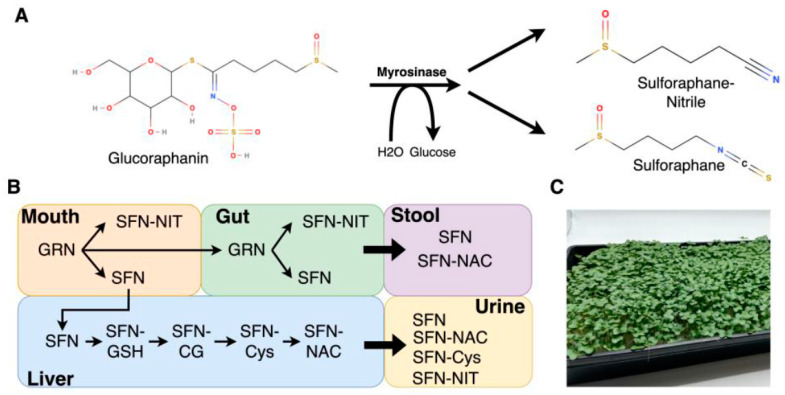
GRN/SFN chemical structures and metabolite secretion schematic. (**A**) Chemical structures of GRN, SFN, and SFN-NIT. (**B**) GRN and SFN metabolism and secretion *. (**C**) Representative picture of broccoli microgreens used in study. * Not shown—SFN metabolites detected in plasma.

Numerous health benefits are associated with the consumption of cruciferous vegetables. A multitude of clinical and preclinical studies suggest cruciferous vegetable consumption can potentially reduce the risk of chronic diseases including cancer, diabetes, cardiovascular disease, and neurological disorders [1,2,3,4]. Cruciferous vegetables (including broccoli, kale, Brussels sprouts, and cabbage) are rich sources of sulfur-containing phytochemicals known as glucosinolates that include glucoraphanin (GRN), glucobrassicin, and sinigrin, among others [5]. Health benefits of cruciferous vegetable consumption are attributed to isothiocyanates that are bioactive hydrolyzed products of glucosinolates. In particular, the isothiocyanate sulforaphane (SFN) which is derived from GRN, the predominant glucosinolate present in broccoli (Figure 1), has been shown in preclinical cell culture and animal studies to exert antioxidant, anti-inflammatory, chemopreventive, and neuroprotective properties [4,6,7]. Furthermore, SFN has also been shown to have synergistic effects in combination with other bioactive phytochemicals, including polyphenols such as curcumin, epigallocatechin-3-gallate, genistein, luteolin, and quercetin [8,9,10,11,12].

Various clinical studies have examined the potential benefits of broccoli consumption and sulforaphane bioavailability using different broccoli sources (mature plants, sprouts, beverages, extracts, and supplements) [13]. Our group and others have focused on the study of broccoli sprouts (3–5 day-old plants) as they contain one of the highest concentrations of GRN compared to mature plants [14]. Of the 92 registered studies related to broccoli, 62 studies involve the use of broccoli sprouts for a wide variety of health conditions and endpoints (clinicaltrials.gov). Another broccoli product that is similarly rich in GRN content but largely unexplored in clinical studies is broccoli microgreens. Microgreens are immature greens harvested 1–3 weeks after germination when the cotyledon is fully developed and before the first true leaves have emerged [15]. Brassicaceae microgreens, particularly broccoli microgreens, are good sources of minerals and antioxidant phytochemicals, including isothiocyanates, and have gained recent interest as promising foods with numerous health benefits [16,17,18]. To date, only one microgreens study (red cabbage and red beet microgreens) is registered on the clinicaltrials.gov database (NCT04239898). To our knowledge, there has not been any human feeding study using broccoli microgreens. It is currently unclear whether the consumption of an equivalent amount of broccoli microgreens will have similar results to broccoli sprouts. There is also a gap in knowledge in regards to whether broccoli microgreens confer similar SFN bioavailability and metabolite profiles compared to broccoli sprouts upon consumption, as reported in other studies.

Another emerging area of study is the relationship between cruciferous vegetables and the gut microbiome. The gut microbiome may potentially play an important role in human health and disease risk [19]. Dietary components are known to alter the abundance and diversity of bacteria in the gut [20]. Clinical trials using broccoli, broccoli sprouts, and other cruciferous vegetables demonstrated modulation of the gut microbiome composition following multiday intervention periods [21,22,23]. Currently, little is known whether broccoli microgreens consumption affect the microbiome. Due to the older age of microgreens compared to broccoli sprouts, they may contain a great amount of fiber and other polyphenols compared to broccoli sprouts—and thus may have a greater impact on the gut microbiome compared to sprouts. Furthermore, we were interested in identifying relationships between variation in the gut microbiome composition and SFN metabolite and excretion profiles in human subjects. Previous studies in broccoli sprouts have shown that variability in SFN-nitrile excretion profiles is associated with specific microbiome profiles [24]. 

The goal of this study was to determine SFN bioavailability and the microbiome profile in healthy subjects fed a single serving of fresh broccoli microgreens, as well as to determine the stability and levels of GRN and SFN in broccoli microgreens over time. We examined urine and stool SFN metabolite profiles and microbiome composition over the course of the two-day study. Our study provides the foundation for future studies that may help elucidate the potential health benefits of broccoli microgreen consumption.

## 2. Materials and Methods

### 2.1. Subjects

Eleven healthy subjects, 22–54 years old (Table 1), were recruited in Corvallis, Oregon to participate in the feeding study that took place between 13 and 15 September 2022. The study was carried out at the Linus Pauling Institute clinical research laboratory and Moore Family Center metabolic kitchen at Oregon State University (OSU). Exclusion criteria included (1) tobacco use; (2) BMI < 18.5 or >30.0 kg/m^2^; (3) pregnancy or breastfeeding; (4) use of oral antibiotic medication within past 6 months; (5) extensive vigorous exercise (7+ h per week); (6) use of medications to control cholesterol or fat absorption; (7) a history of significant acute or chronic illness, bariatric surgery, gastrointestinal procedures or disorders. All participants had provided informed consent and were verified for eligibility. Study protocols were approved by the Institutional Review Board at OSU (IRB #8343). 

### 2.2. Study Design

Subjects were asked to fast overnight. On the first day of study, subjects consumed a single serving of fresh broccoli microgreens (16 g or 0.56 oz) along with a standardized breakfast (bagels and orange juice). Broccoli microgreens (EM variety) were provided by 80 Acres Farms (Hamilton, Ohio). Broccoli microgreens (10 days old) were harvested and shipped overnight on ice to OSU. Upon arrival, microgreens (1 day post-harvest) were kept at 4 °C until study start day (6 days post-harvest). Study start day was chosen to allow for sufficient time to analyze microgreens GRN and SFN content prior to feeding study. Subjects were instructed to avoid consuming foods and beverages containing cruciferous vegetables or live/active cultures, and herbal, phytochemical and probiotic supplements for 1 week before and throughout the two-day study. Self-reported, 9-day diet records were collected (starting 1 week before the study and during the two study days). Diet records were analyzed using Food Processor^®^ SQL (EHSA, Salem, OR, USA). 

### 2.3. Sample Collection

Urine samples: Baseline 0 h spot urine collection was obtained prior to microgreens consumption. Following microgreens consumption, complete urine collections were obtained at 0–3, 3–6, 6–24, and 24–48 h post-consumption. Samples were acidified with boric acid to stabilize SFN metabolites and kept cold throughout the collection periods. Upon receipt, samples were further acidified with trifluoroacetic acid (10% *v*/*v*) and stored at −80 °C.

Stool samples: Stool samples were collected by subjects into (1) CryoELITE^®^ tissue vials (Wheaton, Millville, NJ, USA), and (2) OMNIgene.GUT tubes (DNA Genotek, Ottawa, ON, USA). Samples were collected in the evening or morning before microgreens were consumed (0 h) and before the 24 h and 48 h study visits. Samples were kept cold while in the subjects’ possession and stored at −80 °C upon receipt.

### 2.4. Sample Processing for Mass Spectrometry 

Microgreens: A small sample of fresh broccoli microgreens was immediately processed for mass spectrometry analyses upon shipment arrival (1 day post-harvest) at OSU to determine SFN and GRN contents. A second sample was similarly processed 6 days post-harvest. For SFN measurements, samples were heated to 60 °C in water for 10 min to inhibit epithiospecifier protein activity [25], followed by homogenization and 1 h incubation with 2 mg/mL myrosinase (Sinapis alba thioglucosidase, MilliporeSigma, St. Louis, MO, USA) at 60 °C to fully convert GRN to SFN (SFN equivalence). For GRN measurements, samples were heated to 100 °C in water for 10 min to inactivate all enzyme activities, followed by homogenization, and extracted 3 times in water. SFN and GRN extracts were centrifuged to remove insoluble debris, and soluble homogenates were filtered and further diluted in 0.1% formic acid for analyses.

Urine: Urine samples were centrifuged and filtered to remove protein precipitates and were diluted 1:1 (*v*/*v*) with 0.1% formic acid for SFN metabolite analyses.

Stool: Frozen stool samples stored in tissue vials were partially thawed, a small amount of stool material (~150–200 mg wet weight) was transferred to 2 mL homogenization tubes (Precellys CKMix Tissue Homogenizing Kit, Cayman Chemicals, Ann Arbor, MI, USA) and acidified in 250 μL 70% methanol/10% formic acid/20% water to stabilize SFN in the samples. Acidified samples were dried in a desiccator and weighed. Dried stool materials (~100–150 mg) were resuspended at 1:5 stool:0.1% formic acid (*w*/*v*), and homogenized using Precellys tissue homogenizer (Bertin Technologies, Rockville, MD). Samples were centrifuged and soluble homogenates were filtered for SFN metabolite analyses. 

### 2.5. Mass Spectrometry Analyses

Detection of SFN and SFN metabolites has previously been published [24,26]. Briefly, LC-MS/MS was carried out using a Shimadzu system (Shimadzu, Columbia, MD, USA) coupled to a QTRAP 4000 mass spectrometer (Sciex, Framingham, MA, USA) employing multiple reaction monitoring (MRM) transitions for metabolites detection at the OSU Mass Spectrometry Center. The MRM analysis was conducted in positive ionization mode for SFN and its metabolites. For GRN quantification, the LC method was the same as used in SFN measurements, but MRM analysis was conducted in a negative ionization mode. The following precursor and product ions were used to detect SFN, SFN metabolites, and GRN: SFN (178 > 114), SFN-glutathione (SFN-GSH, 485 > 179), SFN-cysteine (SFN-Cys, 299 > 114), SFN N-acetyl-L-cysteine (SFN-NAC, 341 > 114), SFN-cysteinylglycine (SFN-CG, 356 > 114), SFN-nitrile (SFN-NIT,146 > 98), and GRN (437 > 179). Quantification was determined against known standards. 

### 2.6. Stool Sample Processing for 16S Amplicon Sequencing 

DNA from stool samples collected in OMNIgene-gut tubes was isolated using QIAamp PowerFecal Pro DNA kit (Qiagen, Germantown, MD, USA). PCR was used to amplify the 16S rRNA gene at the V4 region using the Earth Microbiome Project 16S Illumina Amplicon Protocol [27]. Barcoded amplicons were quantitated, pooled, and sequenced using Illumina MiSeq at the Center for Quantitative Life Sciences core facilities at OSU. This approach yielded 300 bp paired-end amplicon sequences at a target sequencing depth of 50,000 reads per sample. Data preprocessing and identification of amplicon sequence variations (ASVs) were carried out using the DADA2 pipeline, as implemented in QIIME 2 (v2022.2) [28]. Briefly, reads were first trimmed for read quality and then filtered for expected errors, followed by a merging of paired reads and removal of chimeric ASVs. Taxonomy was assigned using the readytowear animal distal gut classifier which weighs SILVA taxonomic labels for their relevance to a specific environment [29,30,31].

### 2.7. Microbiome Data Management and Quantification of ASVs

All statistical analysis was conducted in R version 4.2.1, unless otherwise noted. The Benjamini–Hochberg procedure was used for multiple testing correction and an adjusted *p*-value of 0.05 was used to determine significance [32]. Previous work in Bacteroides thetaiotaomicron has shown strain-level differences in GLS hydrolyzing capabilities; thus, we conducted our analysis at the ASV level to capture the microbiome at the most specific level possible. To remove noise from the dataset, sparse ASVs, which were those observed fewer than 2 times with a mean relative abundance across all samples less than 0.0001%, were filtered out of our data set, which yielded a final dataset of 455 ASVs. Rarefaction curves using the vegan package (v2.6-2) in R were built on agglomerated and filtered data to verify that all samples were sufficiently sequenced [33]. 

### 2.8. Microbiome Analysis

The R packages phyloseq (v1.4) and ggplot2 (v3.3.6) were used to visualize and calculate alpha diversity metrics using the unfiltered data [34,35]. Differences in alpha diversity were assessed using Friedman’s test (repeated measured non-parametric one-way ANOVA). Beta diversity of filtered data was analyzed using Principal Coordinate Analysis and based on Jaccard distance [34]. Permutation analysis of variance was conducted using the adonis2 function from the vegan package (v2.6-2) [33]. To identify genera which were differentially abundant across time, we utilized a generalized linear mixed-effect model, as implemented by the package lmerTest [36]. All samples were rarefied to an even depth and data were transformed using the center log ratio (clr) prior to fitting the model. The clr-transformed abundance of each ASV was used as the response variable with discretized time as the predictor variable. One model was built for each ASV. The Benjamini–Hochberg procedure was used to account for multiple tests [32].

## 3. Results and Discussion

Eleven subjects participated in a feeding study at OSU to determine SFN bioavailability and changes in microbiome profile after the consumption of a single serving of broccoli microgreens. Subjects’ characteristics and average caloric and macronutrient intakes are shown in Table 1. Prior to study, microgreens’ GRN and potential SFN contents were tested for GRN/SFN stability at two different time points, chosen to represent when microgreens arrived at OSU (1 d post-harvest), and on study day (6 d post-harvest). There were no significant differences in both GRN (1 d post-harvest: 12.8 ± 0.6 μmol/g, 6 d post-harvest: 13.6 ± 2.2 μmol/g) and SFN (1 d post-harvest: 7.2 ± 0.5 μmol/g, 6 d post-harvest: 6.4 ± 0.4 μmol/g) contents at the two time points tested. Our data showed that broccoli microgreens had a GRN content (~13 μmol/g) that was similar in range to the reported values for broccoli sprouts (16.6 μmol/g) [37], and was stable with cold storage over time. Each subject consumed microgreens containing 100 μmol SFN equivalent. The amount of microgreens consumed was chosen to match SFN content in previous feeding studies using broccoli sprouts [38]. 

SFN is metabolized via the mercapturic acid pathway, resulting in bioactive SFN metabolites, including SFN-GSH, SFN-Cys, SFN-NAC, and SFN-CG, that are distributed in various tissues (Figure 1) [39]. GRN can also convert to SFN-NIT, a biologically inactive metabolite that has recently been shown to be a major metabolite detected in urine and plasma samples after subjects consumed broccoli sprouts [24,38]. The production of SFN-NIT has been shown to be preferred over SFN under acidic conditions, in the presence of iron ions, and in the presence of epithiospecifer proteins [39,40,41]. Additionally, recent work by our group and others has indicated that microbiome composition may also influence the production of SFN-NIT from GRN [42,43,44,45]. Although SFN-NIT has long been known to be produced from GRN, only one study to date has measured it in human urine following the consumption of broccoli sprouts; thus, we were interested in investigating whether excretion profiles were similar in broccoli microgreens [24]. 

**Figure 2:**
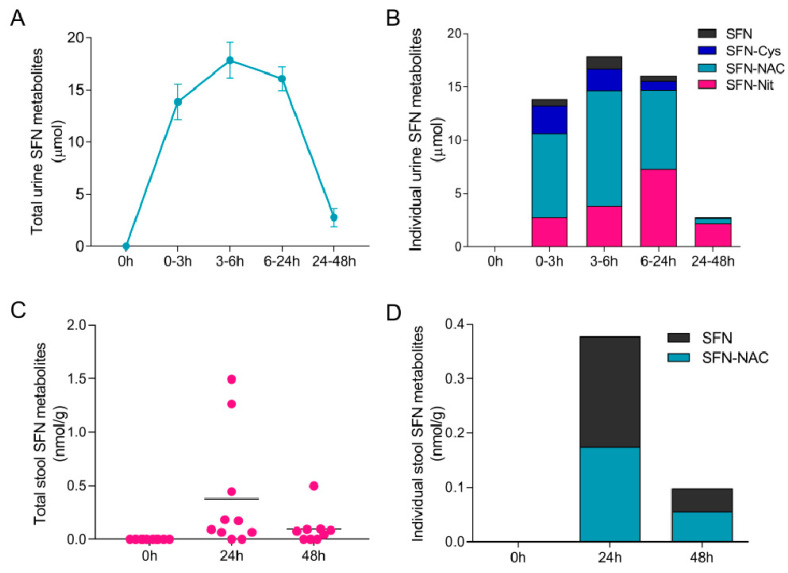
Urine and stool SFN metabolite profile in subjects fed broccoli microgreens. Total SFN metabolites (**A**) and distribution of SFN metabolites (**B**) in urine samples (*n* = 11). Data represent mean ± SEM in (**A**). Total SFN metabolites in individual subjects (**C**) and distribution of SFN metabolites (**D**) in stool samples (*n* = 9–10 due to sample availability). Bars represent mean in (**C**).

We determined SFN bioavailability and metabolite profiles in urine and stool samples in subjects fed a single serving of broccoli microgreens. In urine samples, the excretion of SFN metabolites peaked at 3–6 h post microgreen consumption and a small amount was present in the urine from 24 to 48 h (Figure 2A). SFN and SFN metabolites (SFN-Cys, SFN-NAC, and SFN-NIT) were detected in all subjects, with SFN-NAC being the most abundant at 0–3 and 3–6 h post microgreen consumption (Figure 2B). SFN-NIT excretion peaked at 6–24 h and remained detectable at 24–48 h (Figure 2B). No SFN-CG and SFN-GSH were detected in urine samples. Total mean urine SFN metabolite excretion over the 2-day study was 50.5 ± 2.7 μmol. The urine SFN metabolites profiles in microgreens-fed subjects were similar to those reported previously in broccoli sprouts-fed subjects, including the detection and excretion profile of SFN-nitrile [24,38]. 

To date, the detection of SFN metabolites in stool samples after broccoli sprouts or broccoli sprouts supplement consumption has not been reported. Our current study is the first to report the detection of fecal SFN metabolites after consumption of broccoli products. In stool samples, excretion of SFN metabolites was highly variable among subjects (Figure 2C). SFN and SFN-NAC were detected in 8 out of 10 subjects at 24 h, and 6 out of 9 subjects at 48 h (Figure 2D). No additional SFN metabolites were detected in stool samples tested. Total stool SFN metabolite excretion was 0.57 ± 0.2 nmol/g. While we were able to detect SFN metabolites in human stool, their abundance was much lower compared to what was detected in urine, suggesting stool is only a minor excretion pathway compared to urine. 

The total excreted SFN metabolites was less than the 100 µmols consumed with the broccoli sprouts. Discrepancies was likely attributed to metabolites that were not measured, including SFN metabolites present in plasma (not collected due to study design), as well as inter-conversion of SFN with erucin (ERN), SFN redox partner, which has been shown to vary between human subjects [46]. In vitro studies indicated that some microbes preferentially convert GRN to SFN, while others convert GRN to ERN; thus, differences in microbiome composition may contribute to this variability [42]. Beyond conversion to ERN, the hydrolysis of desulfo-glucosinolates, which may be pre-formed within the microgreens, could contribute to the NIT pool, and some SFN could be conjugated with thiol proteins which were precipitated and discarded [47].

**Figure 3:**
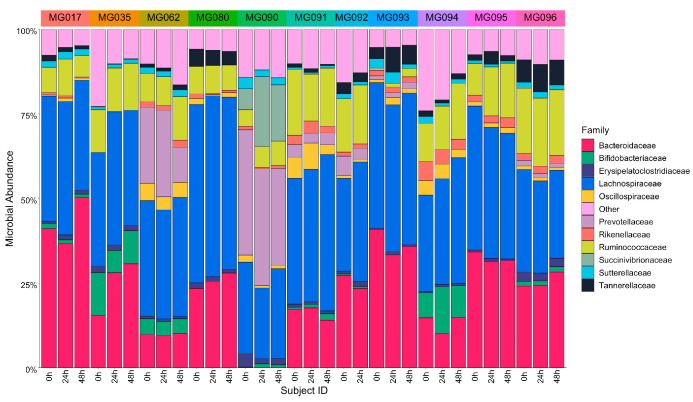
Alpha diversity is not impacted by consumption of a single serving of broccoli microgreens. Relative abundance of microbial families present in the stool of each participant at each time point following consumption of broccoli microgreens (*n* = 11). Top 10 more prevalent families compromising >75% of each sample is shown, the rest are agglomerated into category “other”. Subject ID is listed above, time following consumption of broccoli microgreens below, and the size and color of each bar corresponds to the relative abundance of microbial family, respectively.

**Figure 4:**
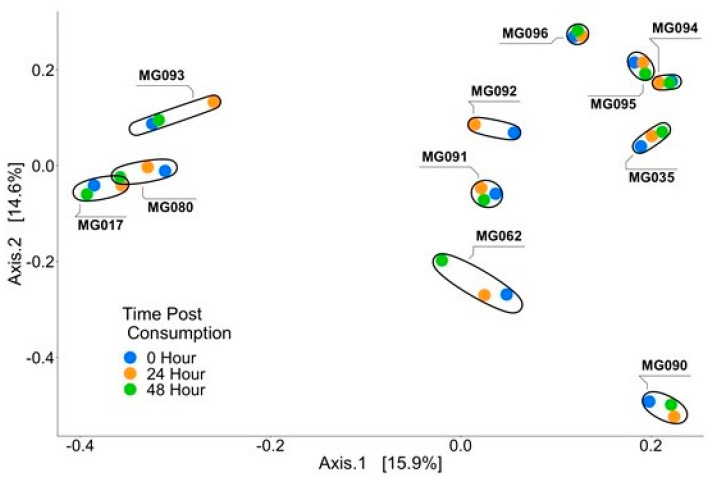
Principal coordinate analysis showing beta diversity of fecal samples from subjects fed broccoli microgreens. Each point represents one sample, with the color of the point indicating the time post consumption of sprouts. All samples from one individual cluster closely together indicating that consumption of broccoli microgreens did not alter beta diversity of the samples.

To determine if consumption of microgreens affects microbiome composition, alpha and beta diversities were evaluated in subjects’ fecal samples. No measure of alpha diversity was significantly different between time points (Figure 3). Similarly, beta diversity was also not significantly different over time (Figure 4). Differential abundance testing was conducted to identify microbes differentially abundant between time points, and no ASVs were found to be differentially abundant across time. Overall, a single serving of broccoli microgreens did not alter microbiome composition. Previous studies have shown that the prolonged consumption of cruciferous vegetables can significantly alter microbiome composition; however, these studies used a dietary intervention period of at least 14 days [21,22,48]. We detected no alterations to the gut microbiome in our study, suggesting a single feeding of microgreens was insufficient to affect microbiome composition. Studies in rodents have shown that the non-glucosinolate components of broccoli are responsible for the gut microbiome modulation observed; thus, it is unclear whether the gut microbiome would shift with the prolonged feeding of microgreens as it does with broccoli [49,50]. Concordant with other studies, we also observed high inter-individual variation in the excretion of SFN metabolites. While the source of this variation is still unknown, it could in part be driven by differences in an individual’s gut microbiome composition. All participants in this study consumed a standardized breakfast with the microgreens, and, thus, differences stemming from dietary components consumed with the microgreens most likely do not explain differences in SFN metabolites. Furthermore, studies examining GST polymorphisms have shown that null-genotypes of GSTM1 and GSTT1 can alter exposure time to SFN metabolites [51,52,53]. However, this would not explain inter-individual differences in the production of SFN from GRN.

One limitation of our work is the short duration of our feeding study, with subjects consuming only one serving of broccoli microgreens, which was not sufficient in modulating microbiome profile. In addition, due to the design of this study, we were not able to directly compare broccoli microgreens to other broccoli products (e.g., broccoli sprouts and supplements). While we were originally interested in identifying relationships between gut microbiome composition and inter-individual differences in gut microbiome composition, due to the relatively small number of participants, we were not able to confidently resolve these relationships. It is also currently unclear whether broccoli microgreens and broccoli sprouts contain similar levels of myrosinase and epithiospecifier protein that can affect SFN content and bioavailability [54]. Future studies with a larger number of participants and longer study duration with repeated consumption will be important in addressing these limitations.

## 4. Conclusions

Significant interest has focused on broccoli sprouts as a dietary intervention due to their exceptionally high content of GRN, the precursor to SFN. This study addresses some of the knowledge gaps relating to broccoli microgreens’ GRN stability, SFN bioavailability upon microgreens consumption, and influence of microgreens on microbiome composition. Our work suggests broccoli microgreens can be a significant source of SFN. The consumption of fresh, unprocessed broccoli microgreens could be a good method for the delivery of bioavailable SFN and SFN metabolites, and potentially confer similar health benefits to broccoli sprouts.

## Figures and Tables

**Table 1:** Participants’ demographics and characteristics.

Demographics ^a^	
Biological Sex	Male (*n* = 4)
	Female (*n* = 7)
Age (years)	35.2 ± 3.2
BMI (kg/m^2^)	23.6 ± 0.7
Race	*n* (%)
White	7 (63)
Non-White	4 (36)
**Average caloric and macronutrient intake ^a.b^**	
Calories (kcal)	1930.1 ± 249
Protein (g)	78.9 ± 12.8
Carbohydrate (g)	232.7 ± 30.7
Fat (g)	76.8 ± 10.6

^a^ Mean ± SEM; ^b^ Average intake based on 9 days of food records.

## Data Availability

The data used to support the findings of this study can be made available by the corresponding author upon request.
